# Case report of recurrent atrial fibrillation induced by thyrotropin-secreting pituitary adenoma with Graves’ disease

**DOI:** 10.1097/MD.0000000000011047

**Published:** 2018-06-15

**Authors:** Jiaqi Li, Huiwen Tan, Juan Huang, Dan Luo, Ying Tang, Ruichao Yu, Hui Huang

**Affiliations:** a Department of Endocrinology and Metabolism; b Department of General Practice; c Department of Pathology, West China Hospital, Sichuan University, Chengdu, Sichuan Province, China; d Department of Pathophysiology and Molecular Pharmacology, Joslin Diabetes Center, Harvard Medical School, Boston, MA, USA.

**Keywords:** Graves’ disease, recurrent atrial fibrillation, thyrotropin-secreting adenoma

## Abstract

**Rationale:**

Thyrotropin-secreting adenoma (TSHoma) is rare. Even though the thyrotoxicosis is mild in patients with TSHoma, it is still a rare cause of arrhythmia, ignore of mild disfunction of thyroid function of TSHoma can lead to the delayed diagnosis of pituitary tumor or leading to recurring of complications. Graves’ disease is an auto-immue endocrinological disorder. Association of TSHoma and Graves's disease is extremely rare. Coexistence of these two diseases made the diagnosis and treatment complicated.

**Patient concerns:**

This patient was a 55-year-old man who had been referred to the department of endocrinology and metabolism of the West China Hospital due to recurrent atrial fibrillation (AF) and thyroxicosis.

**Diagnoses:**

Examinations revealed pituitary thyrotropin-secreting macroadenoma with Graves’ disease.

**Interventions:**

We conducted transsphenoidal surgery. Thyrozol was used to treat the recurrence of Graves’ disease after pituitary surgery.

**Outcomes:**

The TSHoma was successfully cured, and recurrent Graves’ disease was controlled very well.

**Lessons:**

The association of TSHoma and Graves’ disease is extremely rare. Even though the clinical features of thyrotoxicosis are milder in patients with TSHoma, thyroid function tests are still important clinical assessment of patients with AF, which is an arrhythmia associated with hyperthyroidism. TSHoma is a rare cause of thyrotoxicosis; however, ignoring of the mild disfunction caused by TSHoma can lead to the delayed diagnosis of pituitary tumors or to recurring of complications of TSHoma.

## 1 Introduction

Thyrotropin-producing pituitary adenoma (TSHoma) is a rare disease, representing only 1% of all pituitary tumors.^[1]^ The clinical features of thyrotoxicosis are milder in patients with TSHoma, possibly due to a reduction in the biological activity of pituitary-secreted TSH. Even though thyroid function tests to exclude hyperthyroidism are regular clinical assessments of patients with atrial fibrillation (AF), which is an arrhythmia associated with hyperthyroidism,^[2]^ TSHoma is such a rare cause of thyrotoxicosis that the mild thyroid disfunction caused by TSHoma may be ignored, leading to the delayed diagnosis of pituitary tumors or to recurring complications of TSHoma. Graves’ disease is an autoimmune endocrinological disorder. The association of TSHoma with Graves’ disease is extremely rare. The coexistence of these 2 diseases makes diagnosis and treatment complicated. Here, we report a case of a patient with Graves’ disease with recurrent AF induced by TSHoma who experienced a relapse of Graves’ disease 1 year after pituitary surgery.

## 2 Case report

**Figure 1:**
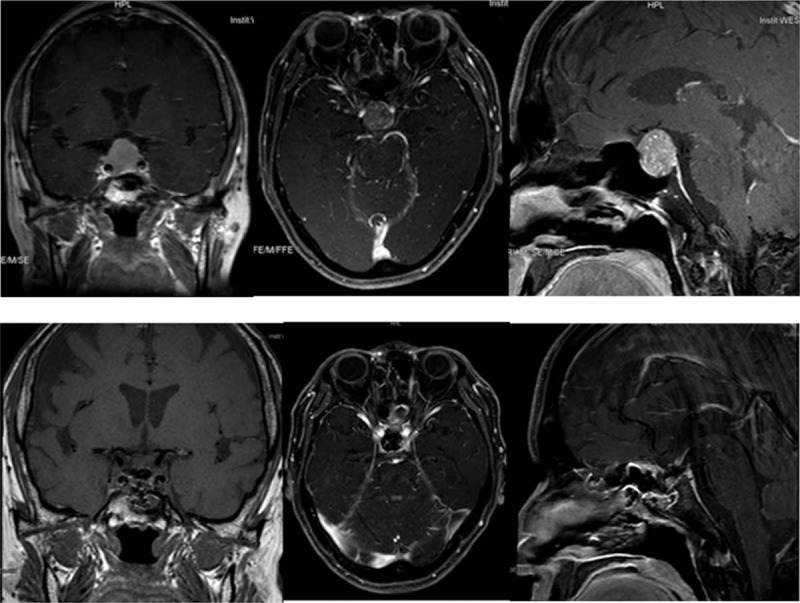
A pituitary tumor measuring 2.3 × 1.7 × 2.2 cm^3^ in the sella, involving the cavernous sinuses and extending into the suprasellar cistern.

**Figure 2:**
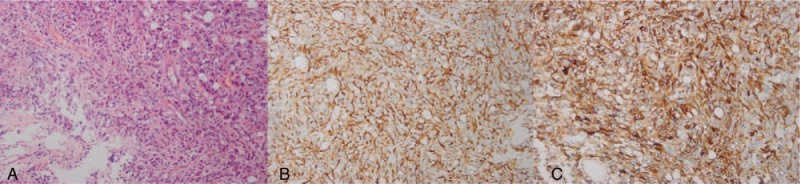
Histopathological findings: Hematoxylin and eosin staining of the cells in the surgical specimen showed the diffuse proliferation of small atypical cells (A, ×200). Immunostaining for thyroid stimulating hormone revealed the expression of TSHb (B, ×200), and the adenoma shows diffuse staining for the antibody against prolactin (C, ×200).

This patient was a 55-year-old man who had consulted at a local hospital for incidentally discovered AF. After being treated with amiodarone for a year, his electrocardiogram (ECG) remained abnormal. Then, he was transferred to the cardiovascular department of our hospital. He had no symptoms such as chest congestion, dizziness, or fatigue, nor did he exhibit symptoms of metabolic syndromes as tachycardia, trembling, or hyperhidrosis. Radiofrequency ablation was performed to treat his AF. The ECG recovered, but the laboratory tests showed TSH 8.9 mU/L (RR, 0.27–4.2), FT3 6.61 pmol/L (RR, 3.6–7.5), and FT4 33.47 pmol/L (RR, 12–22). He ignored the suggestion to consult an endocrinologist. Three months later, the AF recurred, so he received radiofrequency ablation again. Nine months after the patient was discharged from the cardiovascular department, he was referred to our outpatient division for tachycardia, tremors, and thermophobia. At that time, the hormonal examination implied thyrotoxicosis, with a positive result for the TSH receptor antibody (FT3 34.34 pmol/L, FT4 > 100 pmol/L, TSH 0.755 mU/L, TRAb 15.28 IU/L). After administration of thyrozol 10 mg twice a day for 4 months, his symptoms resolved, and his thyroid hormone levels returned to within normal ranges; however, his TSH level was markedly increased. It was suggested to the patient that he should consult an endocrinologist for further examination of the inappropriate secretion of TSH due to central hyperthyroidism. On physical examination at admission, the patient was 172.0 cm tall and weighed 73 kg (body mass index, 24.7 kg/m^2^). His blood pressure was 135/80 mm Hg, and his pulse was 75 beats/min. The thyroid gland was diffusely enlarged, and ultrasonography of the thyroid revealed a rich vascular supply with a nodule located in the left lobe. Fine needle inspiration biopsy suggested the nodule was benign. The computer perimetry suggested a defective visual field. Magnetic resonance imaging (MRI) of the sella region revealed a pituitary tumor measuring 2.3 × 1.7 × 2.2 cm^3^ in the sella, involving the cavernous sinuses and extending into the suprasellar cistern (Fig. 1). Based on these data, we diagnosed the patient with TSH-producing pituitary macroadenoma and central hyperthyroidism. One week after diagnosis, in May 2014, total resection of the pituitary macroadenoma was performed through transsphenoidal neurosurgery. Upon immunohistochemical examination, the resected pituitary adenoma cells exhibited positive staining with the TSH and PRL antibodies, and the percentage of positive Ki-67 was less than 2%, suggesting that the tumor was benign (Fig. 2). After surgery, his TSH levels and thyroid hormone levels remained within the normal ranges. His serum thyroid hormone levels increased beyond the normal ranges 1 year later (TSH 0.223, FT3 11.56, FT4 36.36), although no residual tumor was apparent on the MRI. Positive results were obtained for TRAb, and both TgAb and TPOAb were elevated beyond their normal ranges (TRAb 5.62 IU/L, TgAb 32.79 IU/L, TPOAb 14.13 IU/L). The patient was administered thyrozol 10 mg/day, and his thyroid function tests remained clinically acceptable during 3 years of follow-up. Repeated pituitary MRIs showed a possible mass in the pituitary, suggesting tumor recurrence. In addition, recent ultrasonography showed that the thyroid nodule remained the same size.

## 3 Discussion

TSHomas are usually large tumors with neurological features, and they generally cause enlargement of the thyroid gland. Despite this fact, however, most patients present mild signs of thyrotoxicosis, possibly due to a reduction in the biological activity of the secreted TSH.^[3]^ However, our case illustrates that although uncommon and thought to present with mild symptoms, TSHomas can occasionally cause serious cardiovascular symptoms such as recurrent AF.

In many cases, the correct diagnosis of TSHoma is delayed. In our case, the symptoms and signs of Graves’ disease, such as thyroid goitre with elevated levels of thyroid hormone and TRAb but a decreased TSH level, masked the signs of TSHoma. The diagnosis of TSHoma was made from the presentation due to the inappropriately elevated TSH level after antihyperthyroidism treatment. We reviewed the history of this patient and discovered the previous abnormal results of thyroid function tests and the suspected diagnosis of TSHoma at the first presentation of AF.

Coexistence of TSHoma with Graves’ disease is rare; we searched PubMed for cases of TSHoma associated with Graves’ disease, and only 7 cases have been reported with histological confirmation, all of whom were female.^[4–9]^ This is the first case report of a male patient. In 2 cases, TSHoma occurred before the onset of Graves’ disease,^[4,10]^ as in our case. In the rest of the cases, Graves’ disease coexisted with TSHoma at the initial diagnosis^[11,12]^ or preceded the occurrence of TSHoma.

Elevated levels of free T4 and/or T3 with a normal (nonsuppressed) level of TSH after administration of antithyroid medicine led us to suspect the possibility of a TSHoma and triggered investigations looking for a pituitary cause of the symptoms.

The treatment of TSHoma with Graves’ disease is complicated. In the previous reported cases, antithyroid medication, which was chosen first to treat the Graves’ disease, failed to normalize the FT4, FT3, and TSH levels, resulting instead in the progression of the TSHoma. Administration of antithyroid medication may increase the risk of TSHoma progression due to the positive feedback system.^[13]^ In our case, antithyroid medication was initially administered; however, the TSHoma was successfully treated by endoscopic transsphenoidal neurosurgery. The patient's FT3, FT4, and TSH levels decreased gradually and eventually normalized. However, unfortunately, his FT4, FT3, and TRAb levels increased again and his TSH level decreased 1 year after surgery. At that time, antithyroid medication was administered again without concern for the progression of the TSHoma because the successful removal of the TSHoma also removed the potential risk of tumor enlargement.

The relationship between TSHoma and Graves’ disease is controversial. TSH plays an important role in the maintenance of the normal physiology and immunomodulatory gene expression of the thyroid gland. Therefore, the continuous abnormal hypersecretion of TSH can produce increased levels of antiidiotypic antibodies, causing Graves’ disease. These observations suggest that the normalization of TSH secretion by treating TSHomas may improve Graves’ hyperthyroidism. However, the cause of postoperative recurrence of Graves’ disease in our patient is unclear.

Some reports have shown that Graves’ disease subsequently occurred after TSHoma removal. They suggested that TSH can upregulate the TSH receptor at the mRNA level in cultured human thyroid cells,^[14]^ and it can also suppress the expression of interferon g-induced Fas,^[15]^ intercellular adhesion molecule (ICAM)-1,^[16]^ and class II transactivators on the thyroid cell surface.^[17]^ Therefore, the previous studies suspected that Fas-mediated apoptosis of thyroid cells can be induced by the rapid reduction in TSH levels that occur after tumor resection, which may activate autoimmune responses against the thyroid gland, leading to the occurrence of Graves’ disease after surgery. Hence, the surgical treatment of TSHoma may be considered the first choice in patients with TSHoma associated with Graves’ disease to prevent the enlargement of the tumor induced by antihyperthyroidism therapy.

## 4 Conclusion

We report an extremely rare case of a TSHoma associated with Graves’ disease in a male patient who initially presented with recurrent AF. This case highlights the need to evaluate thyroid function in all patients presenting with AF, and treatment of TSHoma associated with Graves’ disease should be carefully taken into account to avoid the potential risk of tumor progression due to treatment. In addition, physicians should also pay attention to the development of Graves’ disease not only during the progression of TSHoma but also after TSHoma has been treated.

## Acknowledgments

We acknowledge the patient and his family for allowing us to use his medical documentation and information. We acknowledge Dr. Yi Wei of the West China Hospital of Sichuan University for performing the MRI scan.

## Author contributions

**Conceptualization:** Jiaqi Li, Hui Huang.

**Data curation:** Jiaqi Li, Huiwen Tan, Dan Luo, Ying Tang.

**Formal analysis:** Juan Huang, Ruichao Yu.

**Funding acquisition:** Jiaqi Li.

**Investigation:** Jiaqi Li.

**Methodology:** Jiaqi Li.

**Resources:** Huiwen Tan, Dan Luo, Ying Tang, Juan Huang.

**Software:** Dan Luo, Ying Tang.

**Supervision:** Ying Tang.

**Writing – original draft:** Jiaqi Li.

**Writing – review & editing:** Ruichao Yu, Hui Huang.
